# Occurrence and seasonal variation of human *Plasmodium* infection in Punjab Province, Pakistan

**DOI:** 10.1186/s12879-019-4590-2

**Published:** 2019-11-06

**Authors:** Naveeda Akhtar Qureshi, Huma Fatima, Muhammad Afzal, Aamir Ali Khattak, Muhammad Ali Nawaz

**Affiliations:** 1 Department of Animal Science, Faculty of Biological Science, Quaid-i-Azam University, Islamabad, 45320 Pakistan; 2 Department of Medical Laboratory Technology, University of Haripur, Haripur, Khyber Pakhtunkhwa 26220 Pakistan

**Keywords:** *Plasmodium*, Malaria, Incidence, Seasonal variations, Southern and northern Punjab, Pakistan

## Abstract

**Background:**

Malaria is the fifth leading cause of death worldwide. Pakistan is considered as a moderate malaria-endemic country but still, 177 million individuals are at risk of malaria. Roughly 60% of Pakistan’s population, live in malaria-endemic regions. The present study is based upon the survey of various health care centers in 10 major cities of Northern and Southern Punjab to find out the malarial infection patterns in 2015. The diagnosis, seasonal variations, age and gender-wise distribution of *Plasmodium* spp. circulating in the study area were also included in the objectives.

**Methods:**

The malaria-suspected patients ‘16075’ were enrolled for malaria diagnosis using microscopy, out of which 925 were malaria positive which were processed for molecular analysis using nested PCR. The 18S rRNA genes of *Plasmodium* species were amplified, sequenced, blast and the phylogenetic tree was constructed based on sequences using online integrated tool MEGA7.

**Results:**

The 364 cases recruited from Northern Punjab with the highest incidence in Rawalpindi (25.5%) and lowest in Chakwal (15.9%). From Southern Punjab 561 cases were enlisted Rajanpur (21.4%) maximum and lowest from Multan and Rahim Yar Khan (18%). The slide positivity rate, annual parasite incidence, and annual blood examination rates were 5.7 per 1000 population, 0.1, and 0.2% respectively. The only *P. vivax* (66.7%), *P. falciparum* (23.7%) and mixed infection by these two species (9.6%) were diagnosed. The same trend (*P. vivax* > *P. falciparum* > mixed infection) in species identification %age was confirmed from molecular analysis. However, the occurrence of malaria was higher in Southern Punjab (5.5%) as compared to the Northern Punjab (4.0%). The overall malaria percentage occurrence of treatment-seeking patients in all recruited cities of Punjab was 4.9%. The age-group of 1–20 and males among genders were more affected by malaria. The comparison of different seasons showed that the malaria infection was at a peak in Summer and post-monsoon.

**Conclusion:**

The incidence of malaria was high in the flood infected rural areas of Southern Punjab, Summer, and post-monsoon. The age group (1–20) and gender-wise males were more affected by malaria.

## Background

Malaria is the globally largest vector born disease with over 200 million clinical cases happening every year. Malaria has been associated with 0.6 million deaths among children and pregnant women annually. The world is currently not on track to achieve the milestones of 2020 of WHO Global Technical Strategy (GTS) of malaria 2016–2030. The milestone of 2020 is to reduce malaria death and disease by 40%. There is a continuous increase in malaria cases from 2015 to 2018. The number of malaria cases globally increases as 214, 217 and 219 million from 2015, 2016 and 2017 respectively [1–3]. In Pakistan 3.5 million suspected and confirmed malaria cases are reported each year. The history of malaria encapsulates our failure to combat global epidemics as, yet it is the leading threat to public health, economic growth and development in many countries [4]. Malaria is caused by five *Plasmodium* species in human; *P. vivax, P. falciparum, P. ovale, P. malaria and P. knowlesi* (zoonotic infection). Regardless of massive and costly malarial control measurements have been taken over many decades but still, it has re-emerged as a serious health problem in Asia. Sixty percent population of Pakistan lives in malaria-endemic regions. *Plasmodium vivax* contributes 81.3%, *P. falciparum* 14.7%, and mixed-species 4% in the malaria burden of Pakistan [2]. As reported by WHO in 2017 and 2018 [3, 4], Pakistan is among the six WHO Eastern Mediterranean region countries with high malaria transmission and about 100% of the population living at risk. The endemicity of malaria varies in different provinces and even in different cities having variable climates. The cumulative API in all over Pakistan in 2017 was 1.8. Province wise breakdown indicates that during 2017 highest numbers of cases were reported from Khyber Pakhtunkhwa 30%, Sindh 26.5%, Federally Administered Tribal Area (FATA) 21.9%, Baluchistan 20.5% and Punjab with the least epidemiology of 1.1% [2].

The endemicity of malaria varies in Punjab being the land of five rivers Sutlej, Chenab, Beas, Ravi, and Jhelum generate a major breeding ground for malaria vector. Punjab is the most populous province of Pakistan with an estimated population of 110,012,442 according to the 2017 census of Pakistan. It experiences all four types of seasons; spring (March to May), summer (June to September), autumn (October to November) and winter (December to February). The onset of the southwest monsoon in Punjab most likely takes place from the month of June till September but the weather pattern in Pakistan has been irregular since the last few decades. Spring monsoon has either missed or has caused heavy rain over the area that results in floods. The month of June and July are extremely hot. This domineering heat is interrupted by the rainy season in August. The hardest part of the summer season is then over, but cooler weather does not come until late October [5].

Punjab’s plains are low-lying, wet and often swamped. Malaria is seasonal and unpredictable in this province and its epidemics repeated at about 8-year intervals. Variation in malaria transmission from year to year is due to the floods in the Southern Punjab and heavy rainfall during monsoon in Northern Punjab. However, the repeated changes in climate patterns are linked with variation in the malaria transmission pattern [6].

Malaria is more prevalent in rural areas due to low socioeconomic conditions [7]. The prevalence of *P. falciparum* is high among the Afghan refugees than the local population. Several epidemiological studies have revealed that there was a 24 to 36% increase in malaria cases in Pakistan due to the influx of Afghan refugees [7, 8]. The transmission of malaria is seasonal in Pakistan, and a gradual increase in cases can be noted after the July to August monsoon [9]. The *P. vivax* infection is restrained to two peaks per year, the main peak was in late spring as a result relapses of earlier infections and additional peaks occurred in Summer and Autumn by recent transmissions [1]. In contrast to *P. vivax*, *P. falciparum* malaria showed an increasing correlation with August–September temperatures [10]. The wide irrigation network throughout the country, agricultural practices, and monsoon rains provide a favorable environment for the development and growth of malaria vectors [11].

The National Malaria Control Program has reported a six-fold increase in *P. falciparum* infection in the last decade. The increase of *P. falciparum* infection across the country can be associated with chloroquine drug resistance [12] also in this part of the world temperature in Autumn is warmer that increases the transmission rate and due to inappropriate measures of vector control activities [13]. The malaria epidemiology is influenced by environmental factors and socioeconomic conditions that support the vector development and in turn, enhances the parasite and host relationship [14]. Besides these, various other factors such as urbanization, exponential population growth, migration of Afghan refugees, and environmental changes due to excessive monsoon rains, floods, and extensive irrigation projects also favors malarial parasite transmission in Pakistan [1, 15].

The correct diagnosis of *Plasmodium* species is necessary for its treatment and drug formulation. The primary method is microscopy however the most advanced, proficient, sensitive and precise method for the detection of *Plasmodium* species is molecular analysis using Polymerase chain reaction (PCR). The PCR analysis can identify *Plasmodium* species and mixed infections even at low parasitemia [16, 17]. It can also be used to find drug resistance and follow-up therapeutic response [18]. The present study is based upon a complete overview of malaria regarding age, gender and seasonal variations in the two opposite zones of Punjab contributing ten cities. The *Plasmodium* species are also identified circulating in the studied area and their individual incidence is calculated statistically. It will be a great contribution in the epidemiology of malaria in Pakistan and helpful in further malaria control strategies especially in educating people and drug formulation.

## Methods

### Settings, demographic, clinical and laboratory data

**Fig. 1:**
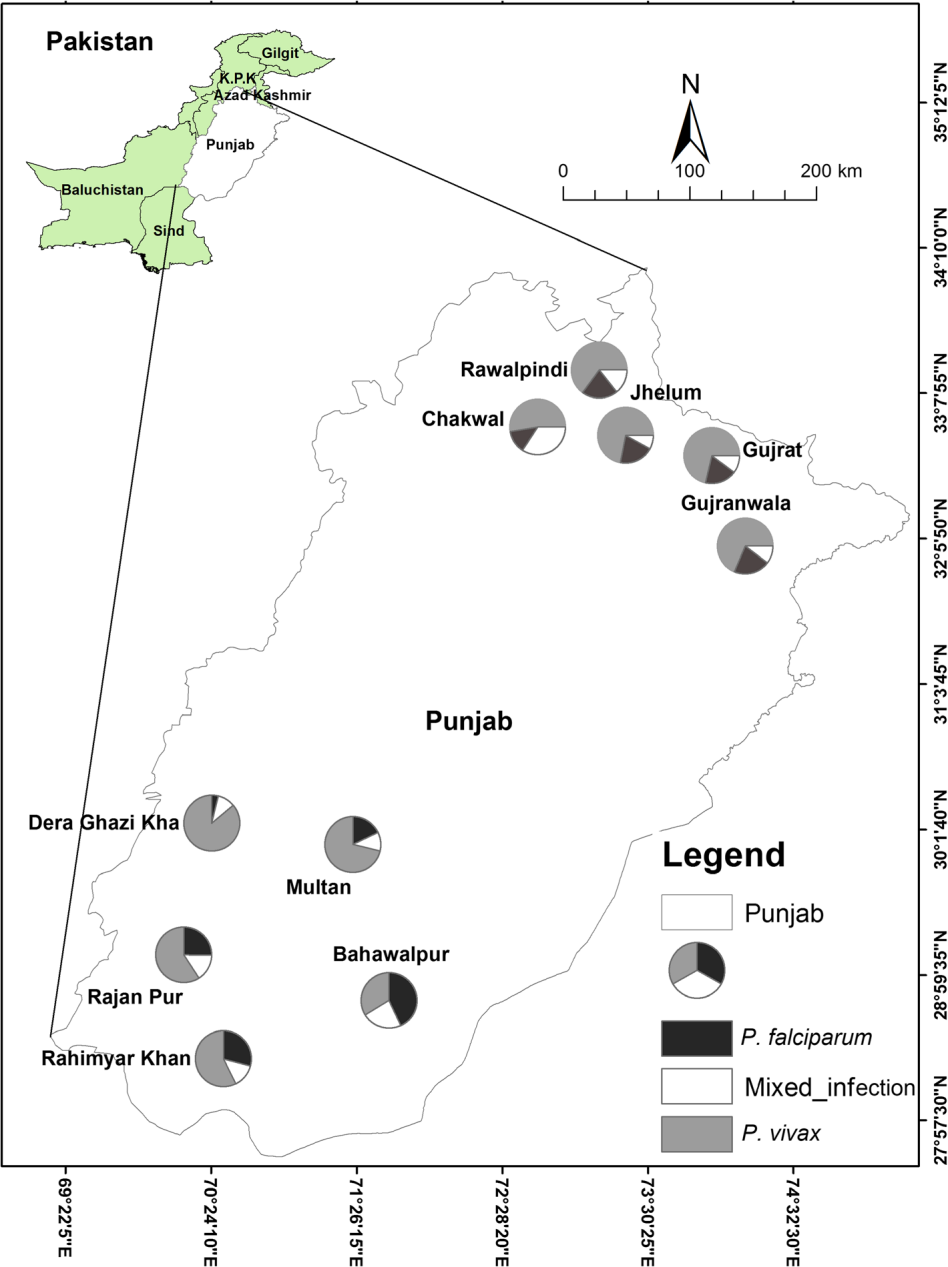
Sample site and proportional distribution of *P. falciparum, P. vivax* and mixed species infection in the cities of Northern and Southern Punjab, Pakistan. The axis shows the coordinates of the respective location in Pakistani map

This study was part of Quaid-i-Azam University Research Fund Pakistan project aiming to determine the in-vivo effect of medicinal plant extracts and nanoparticles on malaria parasite. A cross-sectional epidemiological study of malaria was conducted from January to December 2015, recruited 16,075 suspected malaria cases in different hospitals (see Additional file 1) of 10 major cities of Punjab province from approximately two opposite zones i.e. Northern (Rawalpindi, Gujar Khan, Gujrat, Chakwal and Gujranwala) and Southern Punjab (Dera Ghazi Khan, Rajanpur, Rahim Yar Khan, Multan, and Bahawalpur) (Fig. 1).We collected the data in two following data sets by filling a Performa (see Additional file 2); (i) demographics and baseline characteristics data including gender, age, education, language, location, and occupation, (ii) in clinical data including the malaria symptoms such as shivering, temperature, coma and convulsion. The venous blood (3 mL) was collected in EDTA vacutainers for malaria diagnosis using microscopy and molecular analysis. Microscopy screening was carried out for all suspected cases (16075) and total 925 microscopically positive malaria patient’s blood samples were collected from hospitals to laboratory after taking verbal and written consent (see Additional file 3) from patients/guardians for molecular study, while 15,150 were malaria negative cases. This study was approved and assigned a Protocol (BEC-FBS-QAU-14) by the Bio-Ethical Committee (BEC) of the Quaid-i-Azam University. Slide Positivity Rate (SPR), Annual Parasite Incidence (API) and Annual Blood Examination Rate (ABER) were calculated by different formulas [19] from all suspected cases. The malaria occurrence by molecular analysis was performed according to Khattak et al. [20].

### Molecular analysis

A total of 925 microscopically positive blood samples were selected for PCR confirmation and delivered to Quaid-i-Azam University Parasitology lab in the cold chain. The DNA was extracted by using the Gene Jet Genomic DNA Purification Kit (Thermo Scientific, EU Lithuania), according to the manufacturer’s instructions and then stored at − 20 °C. Human malarial parasitic DNA was detected through nested polymerase chain reaction (PCR) high sensitive detection method was adopted from Snounou et al. [21] using small subunit (SSU) ribosomal RNA gene for *Plasmodium* genus and species (*P. vivax P. falciparum, P. malaria,* and *P. ovale*) detection.

The amplification of the 18S rRNA gene was carried out using the primers and nested cycling conditions reported by Snounou et al. [21]. In a 1st round nested-PCR recipe, 50 μL master mix was used containing; PCR water 32.5 μL, phosphate buffer 5 μL, MgCl_2_ 4 μL, dNTPs 1 μL, 2 μL of each forward and reverse primer, *Taq* polymerase 0.5 μL and 3 μL DNA template. For the 2nd round, 4 μL of the 1st round PCR product was used as a template with the same quantity of master mix as mentioned in the 1st round. A plasmid containing DNA from *Plasmodium* species were obtained from MR4 (Malaria Research and Reference Reagent Resource Center) for the amplification of all five *Plasmodium* species. Few malaria positive samples were also collected from different sentinel sites of the Directorate of Malaria Control Program for positive control whereas, healthy blood samples were used as a negative control. The visualization of amplified PCR products was conducted by electrophoresis using 1.5–2.0% agarose gel stained with ethidium bromide and visualized under UV-trans-illuminator (ExtraGene®, USA). The smear-free amplified PCR products were purified using a gene jet PCR purification kit (Thermo Fisher Scientific, EU Lithuania) (cat# K0701) and sent to Korea (macrogen Inc.) for sequencing. The evolutionary tree was constructed using Mega 7 software.

### Statistical analysis

The count of malarial incidences was analyzed in relation to the age and gender of patients and the season of malaria occurrence. Initial data exploration revealed that count data is zero-inflated and over-dispersed (mean = 9.64 was much lower than variance = 310.31). We, therefore, tested the following three models in R software [22]:

M1: Zero-inflated negative binomial regression with random effect of season.

Zeroinfl (incidences ~ age + gender + species+ 1 | season, data = Disease, dist = “negbin”).

M2: Zero-inflated negative binomial regression without random effect.

Zeroinfl (incidences ~ age + gender + species, data = Disease, dist = “negbin”)).

M3: Simple negative binomial regression.

glm.nb (incidences ~ age + gender + species + season, data = Disease).

For comparison of the models, the Vuong test was used, which suggested that the negative binomial model was a significant improvement (*p* = 1.1672e-06) over a zero-inflated negative binomial model. Therefore, the results of the negative binomial model are used in this study. Data visualization was performed using the ‘ggplot2’ package in the R program. The script for comparison model is given in an Additional file 4.

Cohen ‘Kappa was calculated to measure the level of agreement between two diagnostic tests i.e. PCR and microscopy. The kappa values were categorized as; poor (< 0.2), fair (0.2 to 0.4), moderate (0.40 to 0.6), good (0.61 to 0.80), and very good (0.8) in order to check the strength of agreement between PCR and microscopic examination. The Cohen ‘Kappa tests were conducted on the SPSS V 19.0.

## Results

### Area wise distribution and incidence

Out of 16,075 entire suspected cases, 925 were positive and 15,150 were negative. The overall slide positivity rate (SPR) was 5.7% in all recruited cities of Punjab however; the SPR was higher in Southern Punjab (6.1%) as compared to the Northern Punjab (5.3%). The SPR within the recruited cities of Northern Punjab was highest in Jhelum lowest in Chakwal i.e. 6.8 and 3.1% respectively. In contrast among observed cities of Southern Punjab, the SPR was maximum in Rajanpur (7.1%) minimum in Dera Ghazi Khan (5.4%). The API was 0.1 per 1000 population in all recruited cities.

Similarly, API were also high in Southern Punjab (0.2 per 1000 population) as compared to the Northern Punjab (0.09 per 1000 population). The API within Northern Punjab was high in Jhelum and it was equal to API in Chakwal 0.4 per 1000 lowest in Gujranwala 0.04 per 1000 population. Among the analyzed cities of Southern Punjab, the API was maximum in Rajanpur and minimum in Multan i.e.*,* 1.2 and 0.06 per 1000 population respectively. The ABER was 0.2 in Punjab and it was greater in Southern Punjab as compared to the Northern Punjab 0.26 per 1000 population > 0.2 per 1000 population. The ABER within Northern Punjab was high in Chakwal and minimum in Gujranwala i.e.*,* 1.3 and 0.1% respectively. The ABER in Southern Punjab was highest in Rajanpur (1.7%) lowest in Multan (0.2%) (Table 1).

**Table 1:** Epidemiology of malaria in all recruited cities of Punjab

Sites	Population size	Suspected cases	Microscopic positive cases	Negative cases	SPR (%)	API (per 1000population)	ABER (%)
Northern Punjab	**3,872,437**	**6900**	**364**	**6536**	**5.27**	**0.1**	**0.17**
Gujranwala	2,027,001	1580	80	1500	5.1	0.04	0.1
Gujrat	390,533	930	63	867	6.8	0.2	0.2
Jhelum	174,149	1030	70	960	6.8	0.4	0.6
Chakwal	138,146	1840	58	1782	3.1	0.4	1.3
Rawalpindi	1,142,608	1520	93	1427	6.1	0.08	0.1
Southern Punjab	**3,426,814**	**9175**	**561**	**8614**	**6.1**	**0.2**	**0.3**
Bahawalpur	681,696	1710	119	1591	6.9	0.2	0.2
Rahim Yar Khan	420,419	1895	104	1791	5.5	0.2	0.4
Multan	1,826,546	1770	104	1666	5.9	0.06	0.1
Rajanpur	99,089	1690	120	1570	7.1	1.2	1.7
Dera Ghazi Khan	399,064	2110	114	1996	5.4	0.3	0.5
Total	**7,299,251**	**16,075**	**925**	**15,150**	**5.7**	**0.1**	**0.2**

### Seasonality variations in malaria incidence

**Fig. 2:**
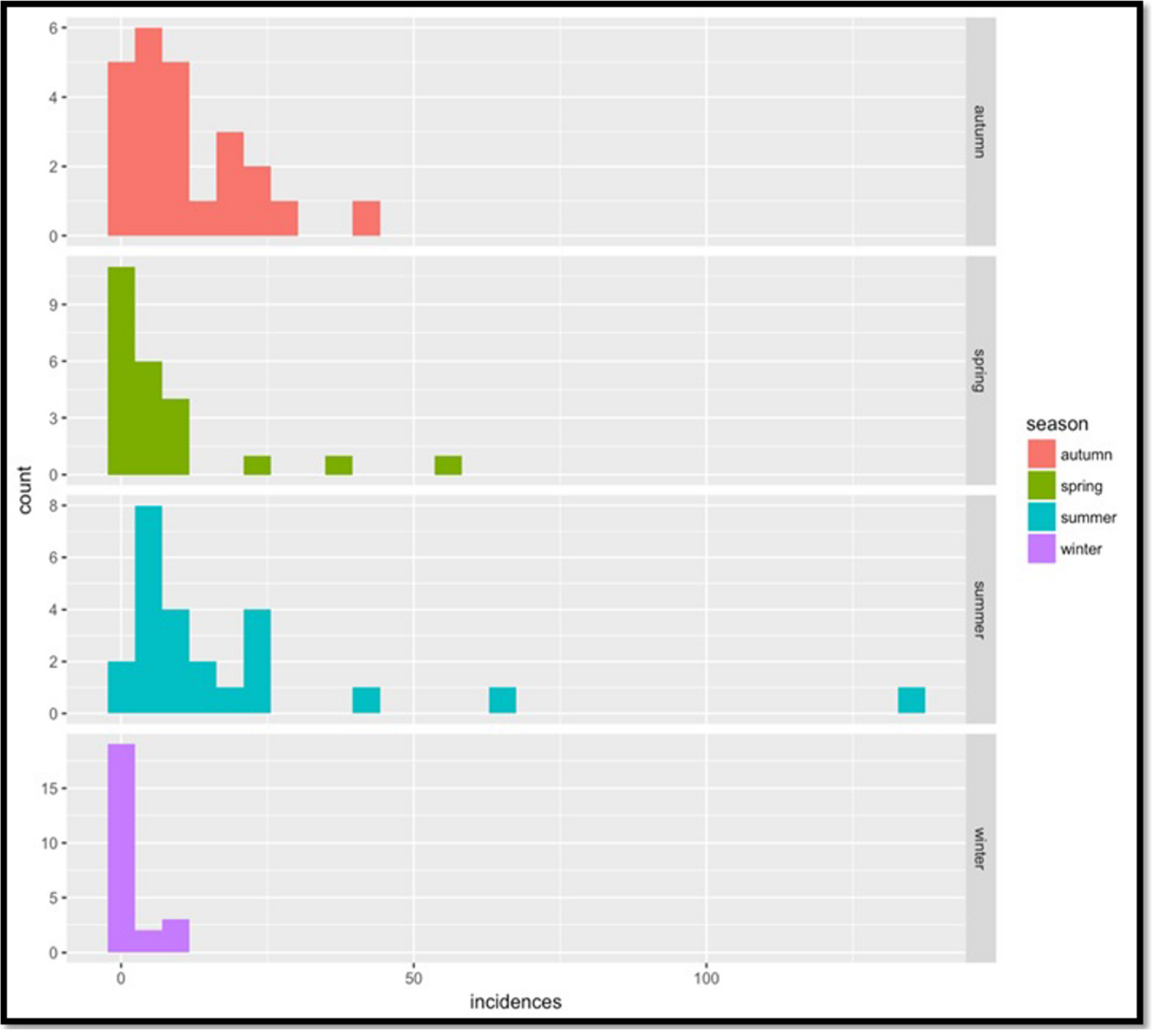
Season-wise distribution of malaria in all recruited cities of Punjab. The x-axis shows the incidence of malarial patients. While y-axis represents the count that is several malaria positive cases based on microscopy. A month is categories into different seasons based on Pakistan metrology

The negative binomial regression analysis (Table 2) indicated a relationship between climate seasonality variation and malarial incidence in Punjab. Results showed the highest malaria parasitemia in summer (June to September) followed by autumn (October to November) and spring (March to May). While low malaria parasitemia was observed during the peak winter dry season (December to February) (Fig. 2). The study area experiences peak rainy season in summer (June to September). At this peak season, highest malaria parasitemia was recorded with expected log (incidence) of 0.3737 higher than that of autumn holding other variables constant**.** Autumn (used as a control in the analysis) had a higher rate of incidences as compared to Spring and Winter with expected incidence (on a log scale) of − 0.5671 and − 2.1374, respectively.

**Table 2:** Negative binomial regression analysis showing effects of age, gender, species and season on incidences of malaria (*N* = 925 spring (March through May), summer (June through September), autumn (October through November) and winter (December through February)

Coefficients	Categories sub type	Estimate	Std. Error	Z value	Pr(>|z|)
Intercept		3.7987	0.2382	15.949	< 2e-16 ***
Age groups	**(1–20) Years**	2.1917	0.1800	12.174	< 2e-16 ***
	**(21–40) Years**	−0.1271	0.1496	−0.849	0.395796
	**(41–60) Years**	−0.9392	0.1653	−5.680	1.34e-08***
	**(61–100) Year**	−2.4390	0.2298	- 10.614	< 2e- 16***
Gender	**Male**	0.8466	0.1256	6.741	1.57e-11 ***
Species Types	***P. vivax*** **&** ***P. falciparum***	−0.8562	0.1751	−4.889	1.01e-06 ***
	***P. vivax***	1.0308	0.1410	7.311	2.65e-13 ***
Seasons	**Spring**	−0.5671	0.1669	−3.397	0.000681 ***
	**Summer**	0.3737	0.1512	2.472	0.013436 *
	**Winter**	−2.1374	0.2311	−9.249	< 2e-16 ***

Significant. Codes: 0 ‘***’ 0.001 ‘**’ 0.01 ‘*’ 0.05 ‘.’ 0.1 “1

The predictor’s age, sex, species and season predicting number of incidences are significant predictors

### Effect of age and gender on malaria incidence

**Fig. 3:**
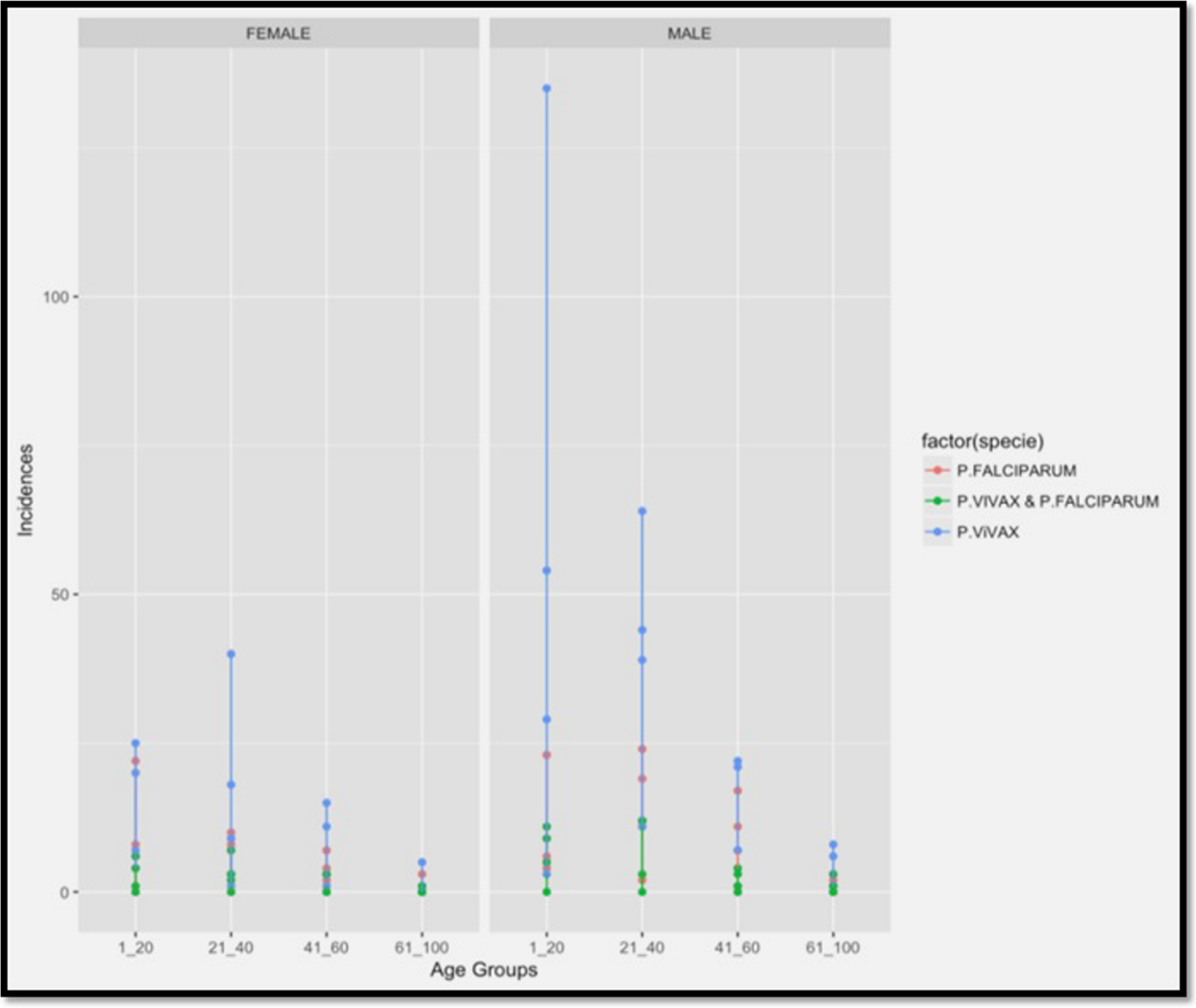
Distribution of *Plasmodium* species in different age groups and in different gender wise based on microscopy from enrolled cities of Punjab

Distribution of malaria case with respect to age and gender are given in the additional file. 5. Age was a strong indicator of malaria occurrence in this study, the young people (< 20 years) with 33.80% were most susceptible to malaria and risk declined gradually in older ages (Table 2). Age group< 20 years is approximately 60% of total population in Punjab Pakistan and mostly involved outside activities. For example, the incidence in the age group 21–40 was − 0.1271 lower (on a log scale) than that of the age group of 1–20 years holding other variables constant. Similarly, incidences in age groups 41–60 and above 60 years were − 0.9392 and − 2.4390 lower, respectively, then that in young people (Table 2, Fig. 3).

Males had an expected log (incidence) of 0.8466 higher than that of females holding other variables constant with 71.78% malaria cases. The *P. vivax* had an expected log (incidence) of 1.0308 higher than that of *P. falciparum*, however, mix infections are − 0.8562 lower than *P. falciparum* (Table 2, Fig. 3).

### Comparative distribution of *Plasmodium* species in the northern and southern Punjab

**Fig. 4:**
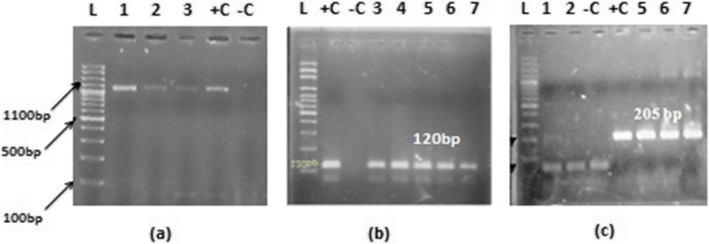
Nested PCR results (**a**) showing the 1100 bp product size of 18sRNA gene of genus *Plasmodium* (**b**) showing the 120 bp product size of 18sRNA gene of *P. vivax* and (**c**) showing the 205 bp product size of 18sRNA gene of *P. falciparum* Lane L: showing 100 bp ladder, Lane +C: showing Positive control of genus *Plasmodium, P. vivax and P. falciparum*, Lane –C: Negative control

The species-wise distribution of malaria, according to the microscopy showed 66.7% (*n* = 617) were *P. vivax* 23.7% (*n* = 219) were *P. falciparum* and 9.6% (*n* = 89) were mixed infections out of 925 recruited cases (Table 3). However, molecular analysis revealed that 53.4% (*n* = 494) as *P. vivax*, 18.7% (*n* = 173) *P. falciparum* and 12.7% (*n* = 119) as mixed species, whereas 15.0% (*n* = 139) were PCR negative cases. However, no case of *P. ovale* and *P. malariae* was found both through microscopy and PCR (Fig. 4).

**Table 3:** Microscopy and PCR based diagnosis of clinical isolates collected from both zones of Punjab

Species	Punjab		Northern Punjab		Southern Punjab	
	Microscopy N (%)	PCR N (%)	Microscopy N (%)	PCR N (%)	Microscopy N (%)	PCR N(%)
*P. vivax*	617 (66.7)	494 (53.4)	270 (74.2)	183 (50.3)	347 (61.9)	311 (55.4)
*P. falciparum*	219 (23.7)	173 (18.7)	69 (19.0)	54 (14.8)	150 (26.7)	119 (21.2)
Mixed (*P. vivax, P. falciparum*)	89 (9.6)	119 (12.7)	25 (6.8)	40 (11)	64 (11.4)	79 (14.1)
Negative	Nil	139 (15.0	Nil	87(23.9)	Nil	52 (9.3)
Total	925	925	364	364	561	561

In Northern Punjab74.2% (*n* = 270) were microscopically positive for *P. vivax*, 19% (*n* = 69) were *P. falciparum* and 6.5% (*n* = 25) had mixed species out of 364 cases. The PCR results showed that 50.3% (*n* = 183) were of *P. vivax*, 14.8% (*n* = 54) *P. falciparum*, 11% (*n* = 40) mixed species and 24% (*n* = 87) did not amplify for any species (Table 3). Similarly, in Southern Punjab 561 cases were recruited. Out of the 347 (61.9%) were microscopically positive for *P. vivax,* 26.7% (*n* = 150) *P. falciparum* and 11.4% (*n* = 64) of mixed species. The PCR results showed that 55.4% (*n* = 311) were of *P. vivax*, 21.2% (*n* = 119) *P. falciparum,* (14.1%) (*n* = 79), mixed species and 9.3% (*n* = 52) PCR negative (Table 3). However, in comparison, the overall incidence was high in the Southern Punjab as compared to Northern Punjab.

The coincidental adjustment of kappa statistics specified that overall agreement in the presence or absence of *Plasmodium* species was good (Kappa = 0.79). However, for the detection of *P. vivax* the agreement between microscopy and PCR was fair (Kappa = 0.38). But for *P. falciparum* and mixed infection, it was moderate (Kappa = 0.53, 0.59) respectively.

### Molecular epidemiology

The average occurrence of treatment-seeking patients in all recruited cities of Punjab was 4.9%. However, the comparison of Northern and Southern Punjab indicated that the occurrence was higher in Southern Punjab (5.5%) as compared to the Northern Punjab (4.0%). Within Northern Punjab, malaria occurrence was higher in Rawalpindi i.e.*,* 5.0% followed by Gujrat 4.9%. The malaria prevalence was lowest in Chakwal i.e. 2.3%. Among the studied cities of southern Punjab, the prevalence followed the pattern Rajanpur (6.7%) > Bahawalpur (5.3%) > Rahim Yar Khan (3.1%), Multan (2.1%) > Dera Ghazi Khan (2.9%) (Table 4)**.**

**Table 4:** Prevalence of *Plasmodium* infection based on PCR results

Localities	Suspected cases	* Malaria occurrence			
		*P. vivax* N (%)	*P. falciparum* N (%)	Mixed infection N (%)	All species N (%)
Northern Punjab	**6900**	**183 (2.65)**	**54 (0.78)**	**40 (0.58)**	**277 (4.0)**
Gujranwala	1580	48 (3.0)	12 (0.7)	8 (0.5)	68 (4.3)
Gujrat	930	30 (3.2)	9 (0.1)	7 (0.7)	46 (4.9)
Jhelum	1030	25 (2.4)	10 (0.9)	9 (0.9)	44 (4.3)
Chakwal	1840	30 (1.6)	8 (0.5)	5 (0.3)	43 (2.3)
Rawalpindi	1520	50 (3.3)	15 (0.13)	11 (0.7)	76 (5.0)
Southern Punjab	**9175**	**311 (3.4)**	**119 (1.3)**	**79 (0.9)**	**509 (5.5)**
Bahawalpur	1710	65 (3.8)	21 (1.2)	14 (0.8)	100 (5.8)
Rahim Yar Khan	1895	59 (3.1)	25 (1.3)	18 (0.9)	102 (5.4)
Multan	1770	54 (3.0)	22 (1.2)	13 (0.7)	89 (5.0)
Rajanpur	1690	70 (4.1)	26 (1.5)	18 (1.1)	114 (6.7)
Dera Ghazi khan	2110	63 (2.9)	25 (1.3)	16 (0.8)	104 (4.9)
All	16,075	**494 (3.2)**	**173 (1.1)**	**119 (0.7)**	**786 (4.9)**

* Malaria occurrence was calculated by dividing the PCR positive cases with suspected cases and multiplied with 100

### Phylogenetic analysis

**Fig. 5:**
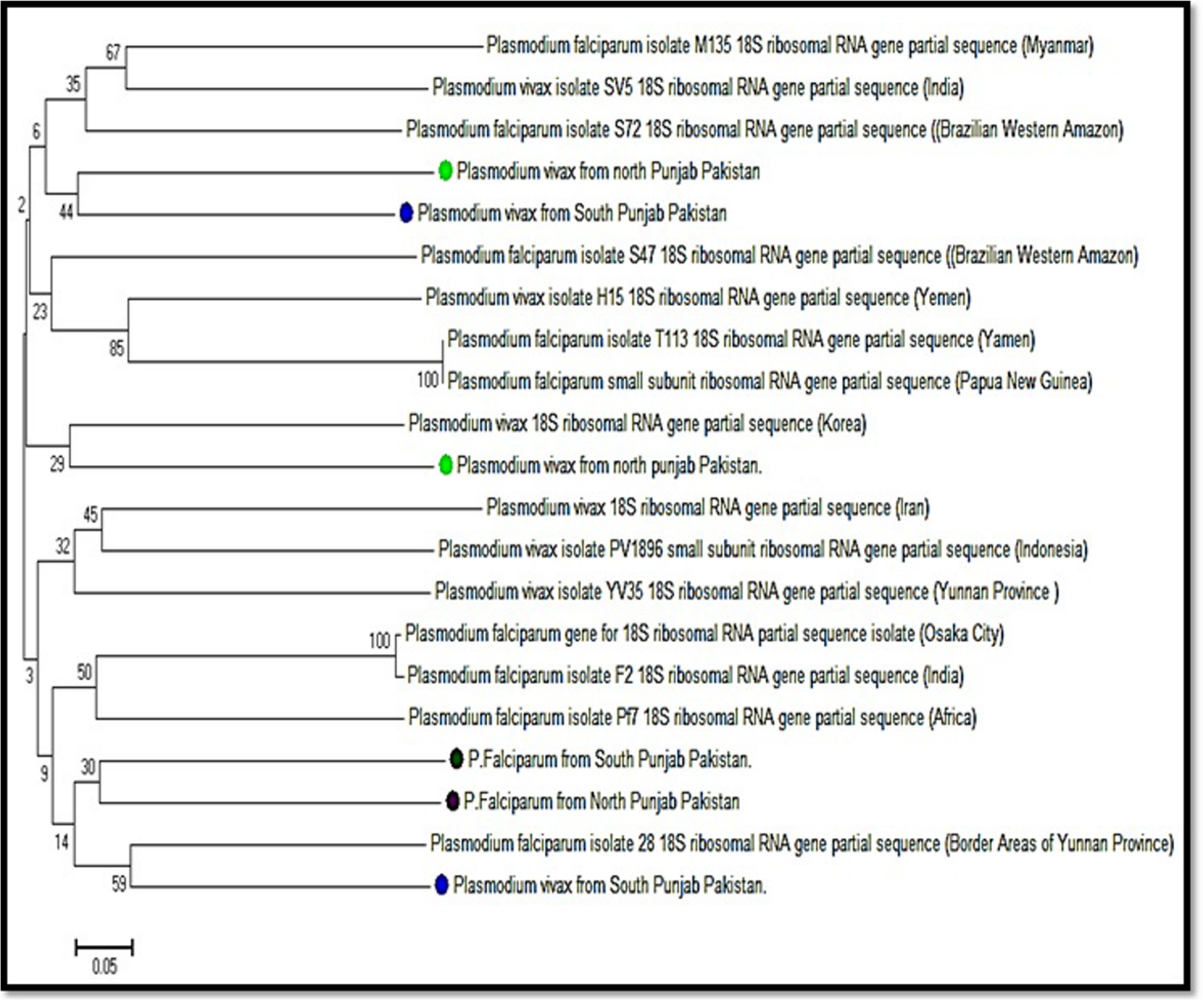
Phylogenetic relationships of *P. vivax* and *P. falciparum* isolates from Northern and Southern Punjab. *Plasmodium vivax* clustered into four and *P. falciparum* clustered into two sub-clusters within the main clade. The neighbor-joining method was applied to infer evolutionary history. The branch length = 6.06796875 was shown and the percentage of replicating trees was shown next to the branches. The p-distance method was used to evaluate evolutionary distance and it was in the units of the number of base differences per site. The analysis involved 21 nucleotides DNA sequences

A phylogenetic tree was constructed based upon sequenced results of both *P. vivax* and *P. falciparum* taken from Northern and Southern Punjab two from each site *P. vivax* and one of *P. falciparum* (Fig. 5)*.* The tree inferred two distinct clades. The *P. vivax* isolates from Northern and Southern Punjab, matched in one group, whereas all the *P. falciparum isolates* from the Northern and Southern Punjab in another clade. Within the main clade, *P. vivax* clustered into four and *P. falciparum* clustered into two sub-clusters. The evolutionary history was inferred using the Neighbor-Joining method. The optimal tree with the sum of branch length = 6.06796875 was shown. The percentage of replicating trees in which the associated taxa clustered together in the bootstrap test (500 replicates) was shown next to the branches. The tree was drawn to scale, with branch lengths in the same units as those of the evolutionary distances used to infer the phylogenetic tree. The evolutionary distances were computed using the p-distance method and it was in the units of the number of base differences per site. The analysis involved 21 nucleotide sequences. All positions containing gaps and missing data were eliminated. There was a total of 85 positions in the final dataset. Evolutionary analyses were conducted in MEGA7. One DNA sequence of *P. vivax* and *P. falciparum* showed the closest relationship with the *P. falciparum* partial sequence of 18S ribosomal RNA gene from Brazilian Western Amazon. Another sample of *P. vivax* from Northern Punjab showed the closest association with the *P. vivax* with a partial sequence of 18S rRNA genes from Korea. However, one sample both of *P. vivax* and *P. falciparum* from Northern and southern Punjab and one sample of *P. vivax* from northern Punjab showed association with *P. vivax* of Yunnan Province (Fig. 5).

## Discussion

Malaria is a serious health problem in Pakistan. Malaria cases vary significantly in different areas and cannot be assessed accurately due to a lack of different information [23]. Pakistan is experiencing all four seasons with extreme weather conditions due to which parasitic species of malaria also have unequal distribution throughout Pakistan and their occurrence changes in seasons. Therefore, it has been difficult to assess the accurate incidence of malaria infection in Pakistan. The occurrence of *Plasmodium* infection can be effectively reduced using active and passive diagnosis [20]. The current study implies on the survey of malaria from 10 endemic cities of Punjab, Pakistan. The WHO in 2014 [24] reported that about 3.3 billion people in 97 countries are at risk of malaria, and 1.2 billion are at high risk with rate of > 1 cases1000 population/year. World Health Organization [25] also reported that approximately 1 million microscopically positive malaria cases in 2010 from the Eastern Mediterranean region out of the 22% were from Pakistan. The definite and accurate estimate of *Plasmodium* infection can also be useful in scaling up malaria surveillance and control measures in Pakistan [13]. In the present study total, 16,075 suspected cases of malaria were ascertained. However, 925 were microscopically positive. Concerning spatiotemporal distribution, more cases were recruited from Northern Punjab as compared to Southern Punjab. The Rawalpindi from the Northern zone and Rajanpur from the Southern zone have the highest malaria cases among all recruited cases.

The overall Slide Positivity Rate (SPR) in two zones of Punjab was 5.7%. However, the SPR was high in Southern Punjab (6.1% > 5.3%) as compared to Northern Punjab. Mahmood et al. [26] studied 348 patients with fever in Karachi and reported 35% SPR. Yasinzai et al. in 2008 [13] reported the SPR between 19.8 to 58.1% among different age groups. Leghari et al. [19] studied the five-year surveillance of Malaria in Bahawalpur city only from 2007 to 2011. They observed SPR from 0.03 to 0.22%. However, in the current study, the SPR was observed as 5.7%. The SPR and API were also high in Southern Punjab 0.2 > 0.1 per 1000 populations as compared to Northern Punjab. Leghari et al. in 2014 [19] found the API in five years from 0.007 to 0.1 per 1000 populations. The ABER was 0.22% in Punjab and it was high in Southern Punjab as compared to the Northern Punjab 0.26 > 0.17%.

In order to prepare for and implement integrated malaria control intervention, it is imperative to understand the seasonality of malaria parasitemia. The outcome of this study confirms that malaria parasitemia in Punjab fluctuates with seasonality variation in climate. In this study, we observed new seasonal patterns by observing the peak prevalence and density of *P. vivax* in post-monsoon season (September through August) which is slightly prolonged from the previous study conducted by Prybylski et al., [27] under a USAID program in Punjab Pakistan. They observed that *P. vivax* was predominant in earlier of the transmission season in July through August. This seasonal shift in *P. vivax* predominance and suppression of *P. falciparum* by *P. vivax* is believed to be related to the greater variation in the climate of Punjab from last decade due to global warming and high production of gametocytemia by *P. vivax* in post-monsoon. Overall malaria began to increase in the spring season i.e.*,* from March and April. These seasonal variations prolonged the summer and increased the highly favorable environmental conditions for mosquitoes breeding that consequently supported the malaria influx in late summer too. However, towards December to February (coldest season) lowest malaria cases occur due to a change in climate, such change in temperature and rainfall patterns unstable the vector dynamics. The age-wise distribution of malaria was also evaluated in the present study. The age of all recruited cases in both genders was ranged between 1 to 78 years. The high incidence was observed among the age group of 1–20 > 21–41 than among 41–60-year age groups. However, the distribution of malaria parasites was low in high age groups. It indicates that the 1–20 age group is highly infected due to low acquired immunity and exposure to vector because of higher non-resting outdoor activities in this age group. Another major factor for highest infection in age group (1–29) is that the major proportion (64%) of total population of Pakistan lies in this age group. The gender-wise distribution indicated that *Plasmodium* infection was dominant in males than females in both zones of Punjab. In the present study, 71% males and 39% females were infected. The females in the Southern Punjab (30.5%) were comparatively high as compared to the Northern Punjab (27.5%). The possible reason may be the role of males in agricultural activities. Khattak et al. [20] reported that males were 64% and females were 36% infected in Punjab. Different reports from different areas of Pakistan showed the males being more affected by malaria infection than females [28, 29], which are comparable to the present findings. This gender-wise variance in malaria transmission is probably due to the socioeconomic norms of Pakistan like the low participation of females in agricultural activities and little access of females to the health care centers and hospitals.

The species-wise distribution of malaria, according to the microscopy showed 66.7% *P. vivax,* 23.7% *P. falciparum* and 9.6% were mixed infections out of 925 recruited cases. However, molecular analysis showed that 53.40% as *P. vivax*, 18.7%, *P. falciparum* and 12.7% as mixed species and 15.3% were PCR negative cases. According to the microscopy the 90 samples were diagnosed as mixed infection, but PCR results showed 119 samples found to have a mixed infection. Many species that were diagnosed as *P. vivax* or *P. falciparum* microscopically had diagnosed as a mixed infection through PCR. The present findings also revealed that mixed infection was high in Southern Punjab as compared to Northern Punjab. Sheikh et al. in 2005 [30] investigated the endemicity of malaria in Quetta, Pakistan from 1994 to 1998 and reported 35% positive smear with 66.8% *P. vivax* and 30.7% *P. falciparum* infection. Likewise, Khattak et al. [20] also reported a malariometric population survey from four province of Pakistan in 2011 and observed *P. vivax* samples were 76%, *P. falciparum* were 18% and mixed infections were 6%. However, they reported 73% *P. vivax*, 22% *P. falciparum* and 5% mixed infection in Punjab province and covered only five cities. While in present study, out of 10 major cities of Punjab highest incidence were found in Rawalpindi 25.5% (*n* = 93) of Northern zone and Rajanpur 21.4% (*n* = 120) from Southern Zone. Ahmed et al. in 2013 [31] also contributed to research on malarial infection from Lal Qilla Lower Dir, Khyber Pakhtunkhwa, Pakistan. They reported malaria infection caused by *P. vivax* as 99.4% and only 0.53% due to *P. falciparum*. Whereas, present study revealed 66.7% as *P. vivax*, 23.7% *P. falciparum* and 9.6% mixed infection (*P. vivax* and *P. falciparum*) by microscopy. The molecular analysis confirmed the presence of the same species with independent and mixed infection although there was a difference in occurrence e.g. *P. vivax*: 53.4%, *P. falciparum*: 16.70% and mixed infection: 12.9%. However, the 139 (15.2%) microscopically positive samples did not amplify with the PCR analysis that may be due to the false-positive results of microscopy or may be due to the DNA damaged by anticoagulant used in EDTA tubes. In contrast, high percentage of *P. falciparum* to *P. vivax* (65% vs 35%) among children positive for malaria has been recorded at Baqai medical university hospital Karachi, Pakistan in 2002 [32]. Mahmood et al. [26] reported a high percentage of *P. falciparum* (88.5%) as compared to *P. vivax* (9%) out of 348 recruited malarial patients in Karachi, Pakistan. Likewise, in Multan, *P. vivax* and *P. falciparum* were observed in a percentage ratio of 60.5:37.2 respectively [33]. The present findings are also in accordance with and following the same pattern in the distribution of *Plasmodium* species from Southern to Northern areas of Punjab province Pakistan, since *P. vivax* infection is becoming higher than *P. falciparum*. It may be due to decrease in temperature and humidity conditions from Southern to Northern Punjab.

With respect to socioeconomic norms, WHO (2010) [34] reported that rural areas of Pakistan were more infected with malaria. The present results also revealed the more occurrence of malaria in Southern Punjab cities due to low development, lack of health care facilities, use of presumptive treatment, as compared to Northern Punjab cities included in the study area which is comparatively more developed to Southern Punjab areas. The environmental factors such as high floods after unprecedented monsoon rains in southern Punjab increase site areas for mosquito vector growth thus increasing malaria incidences. All cities of Southern Punjab indicated that *P. vivax* had a high occurrence except Bahawalpur showed *P. falciparum* as predominant species. Similarly, in Northern Punjab *P. vivax* had the highest prevalence and Chakwal was accounted for the highest mixed infection. The recent flee of IDPs (Internal displacement people) from high malaria prevalence areas such as FATA [35–37] and Swat [13] to Northern Punjab due to war operations against terrorism has also been contributed to the high percentage of *P. vivax* in Northern Punjab. The phylogenetic kinship of *P. vivax* and *P. falciparum* isolates is rather not restricted to one region but seems to be widespread across different geographical regions of the world. The isolates of *P. vivax* from Northern and Southern Punjab showed homology to the *P. vivax* isolates of Yamen and India. All isolates of *P. falciparum* were clustered together and showed homology of *P. falciparum* isolates from Yunnan, Africa, and India. Similar origin of *P. falciparum* was observed by Joy et al. [38]. However, *P. vivax* showed a more diverse population than *P. falciparum.* A significant rise in temperature and humidity result in an increase of vector breeding rate and the vectoral capacity that may enhance the *Plasmodium* infection and its re-emergence [37, 39]. The microscopic observation is a conventional, cheaper and less time-consuming method for the detection of *Plasmodium* sp. However, its accuracy of the results mostly depends upon the experience of technicians along with other factors such as slide staining pattern, parasite level in the blood and microscope condition also influences the results. PCR is used for the most accurate diagnosis of all the five species of *Plasmodium,* but it is expensive than microscopy. The epidemiological study of malaria conducted in the Orakzai agency showed a higher positive result of microscopy as compared to PCR [36, 37]. Shahzadi et al. in 2013 [40] conducted a molecular study for the diagnosis of malaria and found that the PCR based diagnosis is more sensitive, specific and accurate as compared to microscopy that is standard for routine laboratory diagnosis of malaria. In the case of low parasite density and mixed infections, microscopy is not a sensitive test. According to Steenkeste et al. [41], PCR based diagnosis is the best option for large-scale epidemiological studies especially for the diagnosis of mixed infections.

## Conclusion

The malaria control remains a great challenge in developing countries like Pakistan due to agricultural activities; prolong the monsoon season and frequent floods. The relationship between seasonality and *Plasmodium* sp. occurrence in this study exposed that malarial endemicity was highest in summer and post-monsoon season. It might be because of rice paddies and sugarcane cultivation are at peak in this season, which increases the rate of mosquitoes breeding. Along with the effect of seasonal variations on malaria incidence, other contributing factors which may increase malaria endemicity are like misdiagnosis by untrained microscope technician, presumptive treatments, extensive agriculture activities, improper waste management, inadequate health care facilities in remote areas, low socioeconomic conditions and lack of malaria control intervention strategies. The overall incidence rate of malaria in Southern Punjab found to be high as compared to Northern Punjab may be due to the flood in Southern Punjab. *P. vivax* was found to be more dominant as compared to *P. falciparum*. The mixed infection is also very high in Punjab. Pakistan is facing many problems and challenges in the prevention and control of malaria, including misdiagnosis, scarcity of diagnostic facilities, and the use of presumptive treatment. Facts and figures about the burden and species distribution of malaria are acute for guiding national and provincial efforts for effective control measures.

Therefore, it is essential to improve the accuracy of malaria diagnostic techniques specially to determine species-specific and low parasitemia and to assist with diagnostic method procurement decisions in a more professional way. This would also ease the calculation of the ratio of mixed *Plasmodium* species infection and the monitoring of patients receiving anti-malarial treatment. The growing malarial incidence annexed new ecotypes twisted by, industrial growth, and urban development resulting and the green revolution in model shifts towards man-made malaria. The special attention should be given by the government in the flood infected areas of Southern Punjab and many areas of Sindh in order to eradicate malaria from these regions.

## Supplementary information

**Additional file 1.** Location (coordinates) and names of malaria sample collection sites in the province of Punjab.

**Additional file 2.** Performs used for malaria sample and secondary information collection from the suspected cases of malaria.

**Additional file 3.** A parent or gaurdian approval form used to collect malaria blood samples from underage <18 year old malaria patients.

**Additional file 4.** The script used for Zero-inflated Negative Binomial Regression model in R-3.4.4 statistical software.

**Additional file 5.** Number and percentage of malaria cases in whole Punjab with respect to gender and age.

## Data Availability

Additional files hospital names, sampling & consent Performa, age & gender wise malaria distribution and statistical model script are provided in supplementary material along with the manuscript. While the availability of malaria raw data will be provided by the corresponding author on reasonable request.

## Abbreviations

ABER: Annual Blood Examination Rate
API: Annual Parasite Incidence
EDTA: Ethylene Diamine Tetra Acetic Acid
ELISA: Enzyme-Linked Immunosorbent Assay
FATA: Federally Administered Tribal Areas
NCBI: National Center for Biotechnology and Information
RDT: Rapid Diagnostic Test
SPR: Slide Positivity Rate
WHO: World Health Organization
