# Correction to: Occurrence and seasonal variation of human Plasmodium infection in Punjab Province, Pakistan

**DOI:** 10.1186/s12879-020-4767-8

**Published:** 2020-01-08

**Authors:** Naveeda Akhtar Qureshi, Huma Fatima, Muhammad Afzal, Aamer Ali Khattak, Muhammad Ali Nawaz

**Affiliations:** 1 Department of Animal Science, Faculty of Biological Science, Quaid-i-Azam University, Islamabad, 45320 Pakistan; 2 Department of Medical Laboratory Technology, University of Haripur, Haripur, Khyber Pakhtunkhwa 26220 Pakistan

**Correction to: BMC Infect Dis**

**https://doi.org/10.1186/s12879-019-4590-2**

After publication of the original article [1], we were notified that an author’s name has been erroneously spelled. Aamir Ali Khattak should be replaced with Aamer Ali Khattak.
