# Supplementary material for: Occurrence and seasonal variation of human Plasmodium infection in Punjab Province, Pakistan
Source: BMC Infect Dis. 2019 Nov 6;19:935. doi: 10.1186/s12879-019-4590-2 (PMC6836532; doi:10.1186/s12879-019-4590-2)
Supplement: Supplementary file 2 — Additional file 2. Performs used for malaria sample and secondary information collection from the suspected cases of malaria. [file 12879_2019_4590_MOESM2_ESM.pdf]

Performa of sample collection

Date: ____________ Month: ______________ Year :______________

	Subject 1	Subject 2	Subject 3	Subject 4
**Personal**				
**Field visit (s)**				
**Performa No.**				
**Sample I.D**				
**Slide collection**				
Origin of subject				
Gender				
Age				
Language				
Education				
Occupation				
**Contact**				
**Symptoms**				
Shivering				
Temperature				
Coma				
Convulsion				
**Specie Identification (Through Microscopy)**				
P .viavx				
P. falciparum				
P. malariae				
P. ovale				
Mixed infection				
