# Supplementary material for: Occurrence and seasonal variation of human Plasmodium infection in Punjab Province, Pakistan
Source: BMC Infect Dis. 2019 Nov 6;19:935. doi: 10.1186/s12879-019-4590-2 (PMC6836532; doi:10.1186/s12879-019-4590-2)
Supplement: Supplementary file 3 — Additional file 3. A parent or gaurdian approval form used to collect malaria blood samples from underage <18 year old malaria patients. [file 12879_2019_4590_MOESM3_ESM.pdf]

Appendix A

Parental Approval Form

(Parent or Guardian Must Complete)

I hereby give my consent to have my son/daughter__________________ participate in a study about malaria control. I also authorize the physician or technician to share blood sample of my children to Ms. Huma Fatima from Parasitology Lab of Quaid-e-Azam university for research purpose only.

______________________________ ______________

Signature of parent **Date**

______________________________ ______________

Relationship to Child **Telephone**

Address____

City____________________ **State**_____ **Zip code**_______________

Mom/Dad/Guardian's work phone________________________________

Son/Daughter's birth date____________________________________

Name of child's physician____________________________________

Medical History (if any)

____

____

____
