# Supplementary material for: Occurrence and seasonal variation of human Plasmodium infection in Punjab Province, Pakistan
Source: BMC Infect Dis. 2019 Nov 6;19:935. doi: 10.1186/s12879-019-4590-2 (PMC6836532; doi:10.1186/s12879-019-4590-2)
Supplement: Supplementary file 4 — Additional file 4. The script used for Zero-inflated Negative Binomial Regression model in R-3.4.4 statistical software. [file 12879_2019_4590_MOESM4_ESM.docx]

**Script for Zero-inflated Negative Binomial Regression in R software**

rm(list=ls(all=TRUE))

#Disease <- read.table("clipboard", header = T, sep = "\t")

setwd("C:/Users/user1/Desktop/script")

Disease <- read.csv("Disease.rdata.csv", header = T)

#save(Disease , file="Disease.rdata")

##################################################

#Zero-inflated Negative Binomial Regression

##################################################

rm(list=ls(all=TRUE))

require(ggplot2)

require(pscl)

require(MASS)

require(boot)

load("Disease.rdata")

attach(Disease)

colnames(Disease)

summary(Disease)

ggplot(Disease, aes(Incidences)) + geom_histogram()

ggplot(Disease, aes(Incidences)) + geom_histogram() +scale_x_log10()

mean(Incidences)

var(Incidences)

summary(m1 <- zeroinfl(Incidences ~ age + sex + specie+ 1 | season, data = Disease, dist = "negbin"))

summary(m2 <- zeroinfl(Incidences ~ age + sex + specie, data = Disease, dist = "negbin"))

summary(m3 <- glm.nb(Incidences ~ age+sex+specie+season, data = Disease))

vuong(m2, m3)

vuong(m1, m3)

**Script for gg plot in R’**

**Age vs Malaria Incidence**

ggplot(Disease, aes(Incidences, fill = age)) +

geom_histogram() +

# facet_grid(age ~ ., margins=TRUE, scales="free_y")

facet_grid(age ~ ., scales="free_y")

**Sex vs Malaria Incidence**

ggplot(Disease, aes(Incidences, fill = sex)) +

geom_histogram() +

facet_grid(sex ~ ., scales="free_y")

**Species vs Malaria Incidence**

ggplot(Disease, aes(Incidences, fill = specie)) +

geom_histogram() +

facet_grid(specie ~ ., scales="free_y")

**Seasons vs Malaria Incidence**

ggplot(Disease, aes(Incidences, fill = season)) +

geom_histogram() +

facet_grid(season ~. , scales="free_y")

ggplot(Disease, aes(x = age, y = Incidences, colour = factor(specie))) +

geom_point() +

geom_line() +

facet_wrap(~sex) +

labs(x = "Age Groups", y = "Incidences")
