# Supplementary material for: Occurrence and seasonal variation of human Plasmodium infection in Punjab Province, Pakistan
Source: BMC Infect Dis. 2019 Nov 6;19:935. doi: 10.1186/s12879-019-4590-2 (PMC6836532; doi:10.1186/s12879-019-4590-2)
Supplement: Supplementary file 5 — Additional file 5. Number and percentage of malaria cases in whole Punjab with respect to gender and age. [file 12879_2019_4590_MOESM5_ESM.docx]

| Gender | Age | | | | Total | % by Gender |
| --- | --- | --- | --- | --- | --- | --- |
|  | 1-20 | 21-40 | 41-60 | >60 |  |  |
| Male | 224 | 193 | 175 | 72 | 664 | 71.78 |
| Female | 85 | 70 | 81 | 25 | 261 | 28.22 |
| Total | 309 | 263 | 256 | 97 | 925 | 100 |
| % by Age | 33.40 | 28.43 | 27.68 | 10.50 | 100 |  |

**Additional file 5.** Number & percentage of malaria cases in whole Punjab with respect to gender and age.
